# Supplementary material for: Network instability dynamics drive a transient bursting period in the developing hippocampus in vivo
Source: eLife. 2022 Dec 19;11:e82756. doi: 10.7554/eLife.82756 (PMC9762703; doi:10.7554/eLife.82756)
Supplement: Supplementary file 1. — (a) Synopsis of statistical tests related to Figure 1. Numerical data are provided in the Figure 1—source data 1. (b) Synopsis of statistical tests related to Figure 2. Numerical data are provided in the Figure 2—source data 1. (c) Synopsis of statistical tests related to Figure 3. Numerical data are provided in the Figure 3—source data 1. (d) Synopsis of statistical tests related to Figure 4. Numerical data are provided in the Figure 4—source data 1. (e) Synopsis of statistical tests related to Figure 5. Numerical data are provided in the Figure 5—source data 1. (f) Synopsis of statistical tests related to Figure 6 and Figure 6—figure supplement 2. Numerical data are provided in the Figure 6—source data 1. [file elife-82756-supp1.docx]

Supplementary File 1

**Network instability dynamics drive a transient bursting period in the developing hippocampus *in vivo***

Jürgen Graf, Vahid Rahmati, Myrtill Majoros, Otto W. Witte, Christian Geis,
Stefan J. Kiebel, Knut Holthoff and Knut Kirmse

Corresponding author: Knut Kirmse

Email: knut.kirmse@uni-wuerzburg.de

**This file includes:**

Supplementary file 1a–f

**Supplementary file 1a.** Synopsis of statistical tests related to Figure 1. Numerical data are provided in the Figure 1–source data 1.

**Supplementary file 1b.** Synopsis of statistical tests related to Figure 2. Numerical data are provided in the Figure 2–source data 1.

**Supplementary file 1c.** Synopsis of statistical tests related to Figure 3. Numerical data are provided in the Figure 3–source data 1.

**Supplementary file 1d.** Synopsis of statistical tests related to Figure 4. Numerical data are provided in the Figure 4–source data 1.

**Supplementary file 1e.** Synopsis of statistical tests related to Figure 5. Numerical data are provided in the Figure 5–source data 1.

**Supplementary file 1f.** Synopsis of statistical tests related to Figure 6. Numerical data are provided in the Figure 6–source data 1.

**Supplementary file 1f** (continued)
